# Upregulation of T-Cell-Specific Transcription Factor Expression in Pediatric T-Cell Acute Lymphoblastic Leukemia (T-ALL)

**DOI:** 10.5505/tjh.2012.13540

**Published:** 2012-12-05

**Authors:** Müge Sayitoğlu, Yücel Erbilgin, Özden Hatırnaz Ng, İnci Yıldız, Tiraje Celkan, Sema Anak, Ömer Devecioğlu, Gönül Aydoğan, Serap Karaman, Nazan Sarper, Çetin Timur, Ümit Üre, Uğur Özbek

**Affiliations:** 1 İstanbul University, Department of Genetics, Institute of Experimental Medicine, İstanbul, Turkey; 2 İstanbul University, Cerrahpaşa School of Medicine, Department of Pediatric Hematology, İstanbul, Turkey; 3 İstanbul University, İstanbul School of Medicine, Department of Pediatric Hematology, İstanbul, Turkey; 4 Bakırköy Maternity and Children’s Hospital, Department of Pediatrics, İstanbul, Turkey; 5 Ministry of Health Şişli Etfal Teaching Hospital, Department of Pediatric Hematology, İstanbul, Turkey; 6 Kocaeli School of Medicine, Department of Pediatric Hematology, Kocaeli, Turkey; 7 Ministry of Health Göztepe Teaching Hospital, Department of Pediatric Hematology, İstanbul, Turkey; 8 Ministry of Health Bakırköy Sadi Konuk Teaching Hospital, Department of Hematology, İstanbul, Turkey

**Keywords:** T-ALL, Pediatric, Transcription factor, Expression, prognosis

## Abstract

**Objective:** T-cell acute lymphoblastic leukemia (T-ALL) is associated with recurrent chromosomal aberrations andabnormal ectopic gene expression during T-cell development. In order to gain insight into the pathogenesis of T-ALLthis study aimed to measure the level of expression of 7 T-cell oncogenes (LMO2, LYL1, TAL1, TLX1, TLX3, BMI1, andCALM-AF10) in pediatric T-ALL patients

**Material and Methods:** LMO2, LYL1, TLX1, TLX3, BMI1, TAL1, and CALM-AF10 expression was measured usingquantitative real-time PCR in 43 pediatric T-ALL patients.

**Results:** A high level of expression of LMO2, LYL1, TAL1, and BMI1 genes was observed in a large group of T-ALL.Several gene expression signatures indicative of leukemic arrest at specific stages of normal thymocyte development(LYL1 and LMO2) were highly expressed during the cortical and mature stages of T-cell development. Furthermore,upregulated TAL1 and BMI1 expression was observed in all phenotypic subgroups. In all, 6 of the patients had TLX1and TLX3 proto-oncogene expression, which does not occur in normal cells, and none of the patients had CALM-AF10fusion gene transcription. Expression of LYL1 alone and LMO2-LYL1 co-expression were associated with mediastinalinvolvement; however, high-level oncogene expression was not predictive of outcome in the present pediatric T-ALLpatient group, but there was a trend towards a poor prognostic impact of TAL1 and/or LMO2 and/or LYL1 protooncogeneexpression.

**Conclusion:** Poor prognostic impact of TAL1 and/or LMO2 and/or LYL1 proto-oncogene expression indicate the needfor extensive study on oncogenic rearrangement and immunophenotypic markers in T-ALL, and their relationship totreatment outcome.

**Conflict of interest:**None declared.

## Abstract (tr)

**Amaç:** T-hücreli akut lenfoblastik lösemi (T-ALL), tekrarlı kromozom bozuklukları ve T-hücre gelişim basamaklarındaki anormal ektopik gen anlatım profili ile ilişkilidir. T-ALL patogenezine eğilebilmek için, çocukluk çağı T-ALL hastalarında, yedi farklı T-hücre onkogenlerinin anlatım düzeylerini belirlemeyi hedefledik (LMO2, LYL1, TAL1, TLX1, TLX3, BMI1 ve CALM-AF10).

**Gereç ve Yöntemler:** Çocukluk çağı T-ALL hastalarında (n=43) LMO2, LYL1, TLX1, TLX3, BMI1, TAL1, CALM-AF10 gen ekspresyonları kantitatif eş zamanlı PCR yöntemi ile tespit edildi.

**Bulgular:** T-ALL hastalarının büyük çoğunluğunda, artmış LMO2, LYL1, TAL1 ve BMI1 gen anlatımları belirlendi. Normal timosit gelişiminin spesifik basamaklarındaki lösemik tutulumu gösteren genlerden olan LYL1 ve LMO2’ nin artmış ekspresyonu kortikal ve olgun timosit gelişim basamaklarında gözlendi. Bunun yanı sıra, artmış TAL1 ve BMI1 ekspresyonları bütün fenotipik alt gruplarda belirlendi. Altı hastada, normal koşullarda anlatımı görülmeyen TLX1 ve TLX2 gen anlatımları mevcut iken, hastalarımızın hiçbirinde CALM-AF10 füzyon gen transkripti belirlenmedi. Onkogen ekspresyonları ile klinik verileri karşılaştırdığımızda ise LYL1 ve LMO2-LYL1 genlerinin beraber anlatımları mediasten tutulumu ile ilişkili bulundu. Ancak, yüksek onkogen ekspresyonları pediatrik T-ALL grubumuzda hastaların son durumlarının önceden tahmin edilebilmesi açısından anlamlı değildir. Ama TAL1 ve/veya LMO2 ve/veya LYL1 gen ekspresyonlarının kötü prognozla ilişkili olabileceğine dair bir eğilimden bahsedebiliriz.

**Sonuç:** TAL1 ve/veya LMO2 ve/veya LYL1 gen ekspresyonlarının kötü prognozla olan ilişkisi, onkogenik yeniden düzenlenmeler ve immünfenotipik belirteçlerle beraber, daha büyük çalışma gruplarında çalışılması ve hastanın son durum ile olan ilişkisinin ortaya çıkarılması, gelecekteki tedavi protokolleri için önem arz etmektedir.

## INTRODUCTION

T-cell acute lymphoblastic leukemia (T-ALL) is a rare,aggressive malignancy of thymocytes and corresponds to aheterogeneous group of leukemia arrested at various stagesof lymphoid development. T-ALL constitutes 15% of allchildhood ALL and 25% of adult ALL; approximately 30%of patients relapse within the first year of treatment andthe outcome is usually death [1]. T-ALL patients characteristicallyhave recurrent and rare cytogenetic alterationsthat affect the gene expression profile of involved genes.These proto-oncogenes are associated with the pathwaysinvolved in T-cell development, such as differentiation,proliferation, survival, the cell cycle, and self-renewal.Most chromosomal translocations associated with T-ALLresult in juxtaposition of T-cell antigen receptor (TCR) loci(α/δ or β) regulatory elements to proto-oncogenes, thusderegulating expression of the latter [2]. TCR promotersand enhancers are juxtaposed to a number of developmentallyimportant transcription factor genes, includingHOX11/TLX1, TLX3/HOX11L2, TAL1/SCL, TAL2, LYL1,bHLHB1, LMO1, and LMO2. Deregulation of these genesprimarily arrest differentiation at specific stages of T-celldevelopment [3,4,5,6,7].TAL1, TAL2, LYL1, and bHLHB1 genes are part of thebasic helix-loop-helix (bHLH) protein family and functionas transcriptional co-factors that form complexes with E2A/HEB. bHLH protein family members also bind to membersof the LMO gene family. During normal human T-cell development,TAL2 and LMO1 are not expressed, whereasLyl1, Tal1, and Lmo2 expression in mice are restricted tothe earliest double-negative stages of T-cell maturation.TLX1 is a class II homeobox gene normally involved inspleen development that is not activated during normalT-cell development. TLX1-positive T-ALL cases share asimilar gene expression profile characterized by arrest atthe early cortical, CD1-positive thymocyte stage. Translocation/ectopic expression of these transcription factorshave been reported with different percentage in pediatricT-ALL [8,9]. More recently, evidence of NOTCH1 mutationsin 25%-50% of pediatric T-ALL patients has furtherenhanced the biologic heterogeneity of T-ALL [10,11]. Analternative mechanism of increased NOTCH1 activation via loss-of-function mutations of FBWX7 leads to inhibitionof ubiquitin-mediated degradation of the activatedform of NOTCH1 [12].

These transcription factors and their oncogenicity havebeen well documented in mouse models; however, fewof these known oncogenetic markers have been shown tohave prognostic significance in humans. Conflicting out comes have been associated with TLX3 and TAL1 deregulation,and NOTCH1 mutations. TLX1-positive T-ALL patientsfrequently have activating NOTCH1 mutations, andTLX1 over expression and/or translocation confers a betterprognosis, but this association varies by study [13,14,15,16]. Inan earlier study we determined the NOTCH1 and FBXW7mutation status in a pediatric T-ALL cohort [10]. In thepresent study we examined the transcription factor genesLMO2, LYL1, TAL1, TLX1, TLX3, and BMI1, and gene fusionof CALM-AF10 in an effort to identify T-cell-specificoncogenic transcription factors and their association withprognosis in pediatric T-ALL patients.

## MATERIALS AND METHODS

**Participants**

At the time of diagnosis of T-ALL, bone marrow (BM)(n=33) and peripheral blood (PB) (n=10) samples wereobtained from 43 pediatric patients. The study includedpediatric T-ALL patients diagnosed at Istanbul University,Cerrahpaşa School of Medicine (n=15) and IstanbulSchool of Medicine (n=12), Bakirköy Maternity and Children’sHospital (n=5), Ministry of Health Şişli Etfal TeachingHospital (n=5), Kocaeli University, Kocaeli School ofMedicine (n=2), Ministry of Health Göztepe TeachingHospital (n=2), and Ministry of Health Haseki TeachingHospital (n=2). Patients were diagnosed based onFrench-American-British (FAB) Group criteria and theirclinical characteristics are shown in Supplemental [17]. Mean age of the 17 female and 26 male patients was8.8±4.1 years, and the median white blood cell (WBC)count was 68,400x10^9^/L (range: 1300-580,000x109/L).In all, 20 of the patients were in complete remission (CR),14 died, and 9 were lost to follow-up. T-ALL subgroupswere classified according to European Group for the ImmunologicalCharacterization of Leukemias (EGIL) guidelines,as follows: immature: n=16; cortical: n=8; mature:n = 11 [18]. All T-ALL patients received chemotherapy,according to the Turkish ALL-Berlin-Frankfurt-Munster(Turkish BFM) protocol. Patients were already screenedfor NOTCH1 and FBXW7 mutations [10]. Anonymous control thymocyte subsets were obtained from pediatric thymus tissues, using the same guidelinesdescribed for T-ALL subgroup classification [19]. SortedCD34+ CD38– CD1a–, CD34+ CD38+ 1a–, CD34+CD38+ 1a+, ISP (immature single positive), DP (doublepositive) CD3–, DP CD3+, SP (single positive), CD4+,and SP CD8+ cells were used as T-cell stage specific controls.Thymus cells were kindly provided by Dr. Frank J.T.Staal, Erasmus Medical Center, Department of Immunology,Rotterdam, The Netherlands. The Istanbul School ofMedicine Ethics Committee (reference number and date:2008/305 and 20.02.2008) approved the study protocoland informed consent was provided by all the patients. 

**cDNA synthesis and quantitative real-time PCR(QRT-PCR)**

Total RNA was isolated using a Qiagen RNeasy PlusMini Kit (Qiagen, GmbH, Germany), so as to eliminate thegenomic DNA prior to RNA isolation. RNA quality andquantity were measured using a Nanodrop 1000 (ThermoFisher Scientific, Germany), and cDNA was synthesizedfrom 1 μg of total RNA using a random hexamerand MMLV reverse transcriptase, according to the enzymemanufacturer’s instructions (MBI Fermentase Life Sciences,Lithuania). Quantitative real-time PCR (QRT-PCR) wasperformed using an ABI 7700 (Applied Biosytems, FosterCity, CA, USA) with specific primer-probes, as describedby van Grotel et al. [20]. The level of expression of LMO2,LYL1, TLX1, TLX3, BMI1, TAL1, and CALM-AF10 wasnormalized to ABL gene expression. The threshold value,which is the maximum level of expression of each of thenormal thymic subsets, was evaluated for each gene. 

**Statistical analysis**

Relative gene expression was calculated according tothe delta-delta Ct method-based mathematical model[21]. Analyzed gene expression was categorized as highand low, as compared to controls. Categorical variableswere compared using Fisher’s exact test and comparisonof medians was performed using the Mann-Whitney Utest. Remission status was assessed after completion of inductionchemotherapy. Treatment efficacy was analyzedaccording to in vivo response to induction therapy on d33 (<5% BM blasts). Based on the 33-d response, patientswere classified as good (<5% BM blasts) and poor (>5%BM blast) responders. CR was defined as the absence ofleukemic blasts in the peripheral blood and cerebrospinalfluid, <5% lymphoblasts in BM aspiration smears, andno evidence of localized disease. Primary treatment failurewas defined as persistence of PB blasts or ≥25% blasts inBM after induction therapy. Relapse was defined as the reappearance of PB blasts, >5% blasts in BM, or the appearanceof extramedullary manifestations after CR wasachieved. 

The Kaplan-Meier method was used to estimate survivalrates. Median follow-up was 13.28 months (range:1-130 months). Overall survival (OS) was defined as theinterval from the date of diagnosis to the date of last follow-up or death. Relapse-free survival (RFS) was the timefrom the start of CR to the date of analysis or to the firstevent (failure to achieve remission - early death or resistantleukemia-, relapse or death in complete remission). Differenceswere compared using the 2-sided log-rank test. Multivariate survival analysis was estimated according to theCox regression model, and included the variables of geneexpression, gender, age, WBC count, and immunophenotype.The level of statistical significance was set at P=0.05.Statistical analysis was performed using SPSS v.12.0 forWindows (SPSS, Inc, Chicago, IL, USA)

## RESULTS

**Up-regulated oncogene expression found inpediatric T-ALL patients**

**Figure 1:**
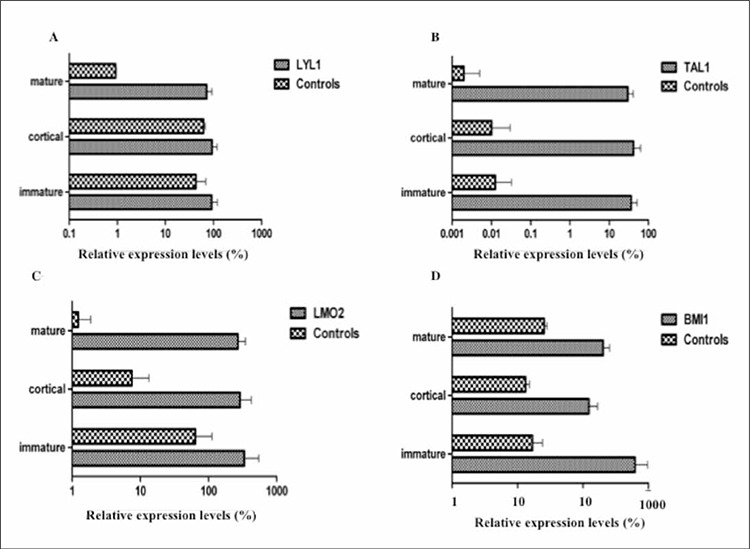
QRT-PCR analysis of oncogenes in the pediatric T-ALL patient subgroups (according to EGIL criteria) and normal thymic control subsets. A. LYL1 expression was significantly higher in the cortical and mature stage subgroups, as compared to their specific controls (cortical versus control: P = 0.02; mature versus control: P = 0.007). B. TAL1 expression was significantly higher in allthe phenotypic subgroups than in the controls (immature versus control: P = 0.01; cortical versus control: P = 0.01; mature versuscontrol; P = 0.02 [Mann-Whitney U test]). C. LMO2 expression was significantly higher in the cortical and mature stage subgroupsthan in the controls (cortical versus control: P = 0.05; mature versus control: P = 0.007). D. BMI1 expression was higher in all thephenotypic subgroups than in their specific controls (immature versus control: P = 0.01; cortical versus control: P = 0.01; mature versus control: P = 0.01).

The patients were grouped according to EGIL criteria(immature, cortical, and mature stages) and the levels of gene expression were compared to normal thymic subsets.A group of patients had an up-regulated gene expressionprofile for LYL1, TAL1, and LMO2. In all, 25 of 41 patients(60.9%) had high-level TAL1 gene expression, 30%of the 43 T-ALL patients had LMO2 overexpression, and10 patients (25.6%) had a high level of LYL1 gene expression,as compared to the controls. Individual gene expressionlevels did not significantly differ between the T-ALLphenotypic subgroups (immature, cortical, and mature).Comparison of the phenotypic subgroups and their stagespecificcounterparts showed that LYL1 expression wassignificantly higher both in the cortical and mature stagesubgroups than in their specific controls (P = 0.02 and P= 0.007, respectively), TAL1 expression was significantlyhigher in all the phenotypic subgroups (immature versuscontrol: P = 0.01; cortical versus control: P = 0.01; matureversus control: P = 0.02 [Mann-Whitney U test]), andLMO2 expression was significantly higher in the corticaland mature stage subgroups (P = 0.05 and P = 0.007, respectively)(Figure 1 A-C). LYL1 and TAL1 co-expressionwas significantly higher in the cortical (P = 0.008 [Mann-Whitney U test) and immature (P = 0.01) stage subgroups. 

Three of the patients had TLX1 expression and 3 othershad TLX3 expression. BMI1 expression was observedin all of the patients and 53.4% of the 43 patients exhibitedover-expression, as compared to the controls. BMI1expression was higher in all the phenotypic subgroups, ascompared to their specific counterparts (immature versuscontrol: P = 0.01; cortical versus control: P = 0.01; matureversus control: P = 0.01 [Mann-Whitney U test]) (Figure1 D). One of the recurrent translocations in T-ALL patientsis t(10;11)(p13;q14-21), which results in the CALM-AF10fusion transcript; none of the pediatric patients were carrying CALM-AF10 fusion. 

**Oncogene expression and outcome in the pediatric T-ALL patients**

To assess the prognostic significance of the above findingswe compared the levels of oncogene expression andpatient clinical characteristics, and then analyzed survivalin the T-ALL patients. High-level LYL1 expression wascorrelated with mediastinal masses (P = 0.01) and otherorgan involvement (P = 0.04). LMO2-LYL1 co-expressionwas strongly associated with mediastinal involvement (P= 0.02 [Fisher’s exact test]). The patients with TLX1 expressionhad mediastinal masses (P = 0.003 [Fisher’s exacttest]) and TLX1 expression was correlated with organinvolvement, including the kidneys and heart P = 0.05[Fisher’s exact test]). TLX3 expression was associated with central nervous system (CNS) involvement (P = 0.04[Fisher’s exact test]). 

**Figure 2:**
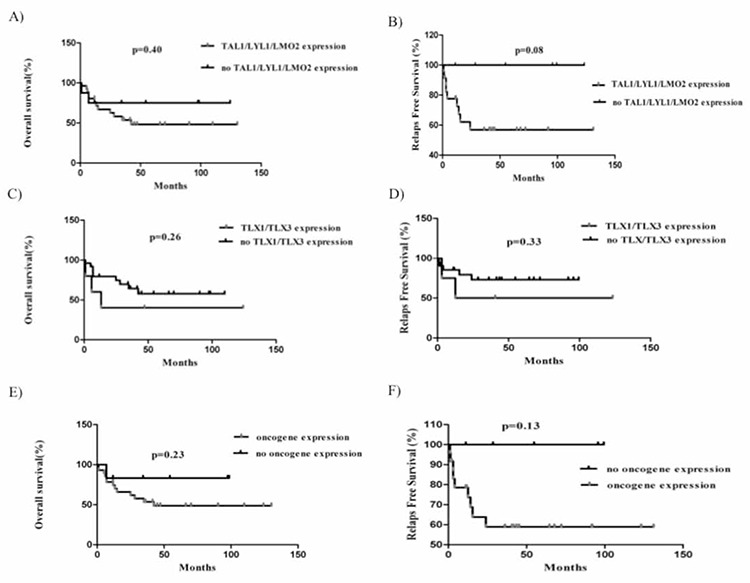
Outcome in pediatric T-ALL patients treated with the Turkish-BFM protocol. A. OS according to TAL1/LYL1/LMO2 expression.B. RFS according to TAL1/LYL1/LMO2 expression. C. OS according to TLX1/TLX3 expression. D. RFS according to TLX1/TLX3 expression. E. OS according to TAL1/LYL1/LMO2/TLX1/TLX3/BMI1 expression. F. RFS according to TAL1/LYL1/LMO2/TLX1/TLX3/BMI1 expression. P value ≤0.05 (two sided) was considered indicative of a statistically significant difference. None of the Kaplan-Meier analyses showed significant differences.

Preliminary comparison of the Kaplan-Meier plotsshowed that there wasn’t a significant difference betweenthe individual oncogene expression groups, except for theTLX3 gene (patients with high-level TLX3 expression versuspatients with no or low-level expression [Cox regression<0.0001; 95% CI: 1.6-18.6]). Additionally, we combinedthe oncogenes, such as TAL1 and/or LMO2 and/orLYL1, TLX1 and TLX3, TAL1 and/or LMO2 and/or LYL1and/or TLX1, and TLX3 and/or BMI1. The outcome ofthese synergistic subgroups versus patients with normalexpression of the oncogenes did not reach the level of statisticalsignificance (Figure 2); however, the obtained datashowed a trend toward longer RFS in the patients with normalTAL1 and/or LMO2 and/or LYL1 expression (P = 0.08[Mantel-Cox test]). NOTCH1/FBXW7 mutations were observedin all the genetic subgroups. Although significant differences were not observed inRFS or OS between the NOTCH1/FBXW7 wild-type andmutated cases, as previously described, we noted a trendtoward longer RFS in the mutant patients (Supplemental Figure 1) [10]. Hence, we considered NOTCH1/FBXW7mutation to be a marker of good prognosis and re-analyzedthe survival data for the oncogene expressions. As aresult, we observed an independent correlation between ahigh oncogene expression profile and short OS and RFSboth in NOTCH1/FBXW7 mutants and wild-type groups,even it did not show a significant difference.

## DISCUSSION

Deregulation of signaling pathways that control normalT-cell development in the thymus plays a crucial rolein T-ALL leukemogenesis. These pathways—under normalcircumstances—are strictly regulated by transcriptionfactors, which are also proto-oncogenic proteins. A fewmolecular mechanisms suggested for T-ALL pathogenesisincluding the mutations in NOTCH1 and FBXW7 genesleading to NOTCH pathway activation and ectopic expressionsof the specific transcription factors such as LYL1,TAL1, LMO2, TLX genes. Currently, there are no geneticmarkers that can be used to reliably predict treatment responseand/or outcome in pediatric T-ALL patients [22].

LMO2, LYL1, and TAL1 genes are members of thebHLH protein family. Among the phenotypic subgroupsin the present study (immature, cortical, and mature),LYL1 and LMO2 exhibited the highest level of expressionin immature cases, as reported by Meijerink et al. [23]. 

Recognition of specific T-cell developmental subgroupsmay have prognostic relevance in T-ALL. Among pediatricT-ALL patients, a pro-T immunophenotype was stronglycorrelated with poorer outcome than other T-cell phenotypes[24]. In contrast, comparison of LYL1 and LMO2gene expression with their specific counterparts in thepresent study showed that the most significant differencesoccurred in mature stage cases. Moreover, LYL1 alone andLMO2-LYL1 co-expression were strongly associated withmediastinal involvement. We classified gene expression in the cases as TAL1/LYL1/LMO2 up-regulated or normal(Figure 2A and B), and analyzed the Kaplan-Meier estimatesof OS and RFS. Although it was not significant butTAL1/LYL1/LMO2 up-regulated patients showed poorRFS rates. To date, it is unclear if TAL1/LYL1/LMO2 expressioncan be used to predict treatment outcome, althoughsome studies suggest that it can [25]. 

The CALM-AF10 (MLLT10) fusion gene t(10;11)(p13;q21) is a common transcript in acute leukemia. Ithas been reported that 9% of adult T-ALL patients have CALM-AF10 fusion and it is restricted to immature lineage[26]. CALM-AF10-positive ALL is associated with TLXfamily members and their transcriptional regulator, BMI1[26]. The BMI1 gene determines the proliferation capacityof normal and leukemic stem cells [27]. In the presentstudy none of the T-ALL patients carried the CALM-AF10fusion gene, whereas 53.4% of the cases had elevatedBMI1 mRNA levels. Increased BMI1 expression in pediatricT-ALL cases is generally ectopic and independent ofCALM-AF10 fusion. It was also suggested that up-regulatedexpression of BMI1 is responsible for the aggressivenature of T-ALL. Although a specific relationship betweenthe present patient’s clinical features and high-level BMI1expression was not observed, T-ALL is the most aggressiveform of ALL. 

TLX genes are normally not expressed in adult tissues[28]. Approximately 7%-20% of childhood T-ALL patientshave ectopic TLX1 and TLX3 expression [15,16]. Inthe present study 12% of the patients had ectopic expressionof TLX1 and TLX3. Some studies indicate that ectopicTLX1 or TLX3 expression confers a poor response totreatment, whereas others report that they do not [5,13].All the present study’s patients with TLX1 expression hadmediastinal masses and those with TLX3 expression hadsignificant CNS involvement, which may consider a possibilityof poor prognosis. TLX gene expression may figureout TLX1 or TLX3 translocations but unfortunately thesepatients lack of cytogenetic data to validate. 

NOTCH1 activating mutations occur in 30%-60% ofT-ALL patients [10,11]. Additionally, inactivating mutationsin the E3-ubiquitin ligase gene FBXW7 contribute toaberrant expression of NOTCH1. TLX1 and NOTCH wasreported to be synergistically activated to regulate transcriptionin T-ALL [29]. These mutations were observedin all the present study’s genetic subgroups, but weren’tcorrelated with oncogene expression or OS ans RFS, butthe number of patients with TLX1 expression was insufficientfor reaching any conclusion. 

The present findings offer some clues about the effectsof activated transcription factors in pediatric T-cellleukemogenesis, prognostic parameters, and therapeuticapplications in different thymic subsets. The most significantfinding of the present study is that deregulation ofmultiple transcription factors (LMO2, LYL1, TAL1, TLX1,TLX3, and BMI1) was involved in the differentiation of Tcells,which is in agreement with other reports [1,2,3,4,5]. Thepresent findings are relevant to 3 main topics. The firstis the etiological point of view that the development ofpediatric T-ALL is associated with up-regulation of several oncogenic transcription factors in a stage-specific manner.T-cell-specific oncogene expression has a greater impactin mature stage patients than in immature stage patients.The second is the prognostic implication of which identificationand validation of oncogenic transcription factorsin T-cell leukemia may lead to the development of newprognostic markers; these may then be useful for patientfollow-up in the future. Lastly, identification of specificexpression profiles in pediatric T-ALL subgroups may aidthe development of new therapeutic applications and protocols. 

**Acknowledgments**

This study was funded by the Scientific and TechnologicalResearch Council of Turkey (TÜBİTAK) (Projectno: 106S112);

**Conflict of interest statement**

The authors of this paper have no conflicts of interest,including specific financial interests, relationships, and/or affiliations, relevant to the subject matter or materialsincluded.
